# Construction and verification of a histone deacetylases-related prognostic signature model for colon cancer

**DOI:** 10.1038/s41598-024-59724-x

**Published:** 2024-04-18

**Authors:** Lei Hao, Weiqi Lu, Jianyu Wu, Yuzhong Chen, Dongni Xu, Peizong Wang

**Affiliations:** 1 Thyroid Hernia Surgery, The First Affiliated Hospital of Guangzhou University of Traditional Chinese Medicine, Guangzhou, 510405 People’s Republic of China; 2 State Key Laboratory of Oncology in South China, Guangdong Key Laboratory of Nasopharyngeal Carcinoma Diagnosis and Therapy, Guangdong Provincial Clinical Research Center for Cancer, Sun Yat-sen University Cancer Center, Guangzhou, 510060 People’s Republic of China; 3 Department of Anesthesiology, Sun Yat-Sen Memorial Hospital, Sun Yat-sen University, Guangzhou, 510120 People’s Republic of China

**Keywords:** Colon adenocarcinoma, Histone deacetylases, Signature, Bioinformatics, Immunotherapy, Cancer, Cell biology, Computational biology and bioinformatics, Gastroenterology

## Abstract

Histone deacetylases (HDACs) contribute significantly to the initiation, progression, and prognosis of colorectal adenocarcinoma (COAD). Additionally, HDACs regulate the tumor microenvironment, immune escape, and tumor stem cells, and are closely linked to COAD prognosis. We developed a prognostic model for COAD that incorporates HDACs to evaluate their specific roles. The COAD dataset containing clinical and mutation data was collected using the TCGA and GEO databases to obtain genes associated with HDAC. LASSO analysis and univariate and multivariate Cox regression analysis were used to determine the presence of prognostic genes. Multivariate Cox analysis was also used to determine risk scores for HDAC-related features. Furthermore, genomic alterations, immune infiltration, and drug response were compared between high- and low-risk groups. Cellular experiments validated the potential regulatory role of BRD3 on COAD proliferation, migration, and apoptosis. The median risk scores, calculated based on the characteristics, demonstrated a more significant prognostic improvement in patients in the low-risk group. Furthermore, HDAC-related features were identified as important independent prognostic factors for patients with COAD. Additionally, genomic mutation status, immune infiltration, and function, as well as response to immunotherapy and chemotherapy, were found to be associated with risk scores. Subgroup analyses indicate that anti-PD-1 therapy may be beneficial for patients in the low-risk group. Additionally, a decrease in risk score was associated with a decrease in immune infiltration. Finally, HCT116 and HT29 cells exhibited inhibition of BRD3 gene proliferation and migration, as well as promotion of apoptosis. In patients with COAD, HDAC-related characteristics may be useful in predicting survival and selecting treatment.

## Introduction

The incidence of colon adenocarcinoma (COAD) is increasing at a rate of more than 4% per year, making it the third most prevalent malignancy^1,2^. Colon adenocarcinoma is the 4th leading cause of death worldwide and is an important public health problem^3^. However, the 5-year survival rate of patients treated with chemotherapy, radiotherapy, and surgery is only about 64%, and the metastasis and recurrence rates of COAD remain high^4,5^. Therefore, the identification of molecular biomarkers can be used to detect early recurrence or minimal residual impairment^6^. Currently, the construction of molecular risk classifications emphasizes the exploration of both prognostic and therapeutic aspects of COAD, thus deepening the understanding of its underlying mechanisms^7^.

It is the sequence and epigenetic modifications of nucleotides that determine the fate and homeostasis of a cell^8^. The growth and differentiation of cells are controlled by the functions of dynamic acetylation and deacetylation cycles^9^. Histone deacetylases (HDACs) are histone acetyltransferases (HATs) that govern the state of protein lysine acetylation to counteract nuclear and cytoplasmic signaling^10^. HDACs play important roles in cancer cells, such as regulating gene expression, inhibiting apoptosis and promoting cell proliferation, modulating DNA damage repair mechanisms, and the activity of transcription factors and nuclear receptors^11^. Among other things, HDAC2 can control proapoptotic signals in COADs if it acts as a modifier of aberrant Wnt pathway activation^12^. Inhibitors of HDAC (HDACi) can be utilized for treating refractory cutaneous T-cell lymphoma, indicating the possibility of helping COAD patients by targeting HDAC family members^11^.

Overall, HDACs may play a critical role in COAD and may serve as novel targets for therapy. Therefore, we established HDAC-associated signatures to evaluate their potential role in COAD patients and to provide better prognostic tools for COAD patients. Specifically, we generated a feature set containing five genes that were highly correlated with HDAC by comprehensively analyzing gene expression data in tumor tissue samples. We used these features to calculate a risk score for each COAD patient and then categorized the patients into a high-risk group and a low-risk group. Our study was also a comparison of genomic alterations and immune infiltration between these two groups of patients. TMB level and CNV signature profiles provide mutational load and genomic structural variation to assess tumor response and prognosis. Immunoprofiling was performed to assess the types and numbers of immune cells in the tumor microenvironment and the expression levels of immune-related genes. By analyzing the relationship between genomic alterations and immune infiltration profiles, we hope to gain insight into the biological differences between the high-risk and low-risk groups and how these differences affect the development of COAD and treatment response. Thus, our study aims to provide new insights into diagnostic and therapeutic strategies for COAD and lay the foundation for the development of more effective treatments.

## Methods

### Data acquisition and analysis of gene expression levels related to HDAC-related gene

We downloaded RNA expression profiles, mutations, and clinical data from the TCGA database (https://portal.gdc.cancer.gov) for the colorectal cancer cohort (COAD). We collected data from 418 patients and excluded patients with a survival of less than 30 days. RNA expression data were converted to TPM format for subsequent analysis. For external validation, we obtained expression profiles of the GSE17538 cohort from the GEO database (https://www.ncbi.nlm.nih.gov). We performed a correlation analysis between the expression level of each HDAC-related gene and clinical characteristics, such as the age of the patients, the tumor stage (T-N-M), the staging, and the survival time. In addition, we evaluated and ranked the HDAC levels of each sample for further analysis.

### Construction of HDAC-related signature

We first used univariate Cox analysis to identify genes associated with prognosis. Then, we used LASSO analysis to identify prognostic genes of value and excluded genes of no value. Next, we evaluated the regression coefficients of genes of value in each COAD patient and the risk scores associated with them using multivariate Cox regression analysis. We categorized patients into high-risk and low-risk groups based on the median risk score. The overall survival (OS) of patients in the two groups was compared. Kaplan-Meyer (KM) curves were used. We also determined the validity and accuracy of risk scores in predicting 1-, 3-, and 5-year prognosis using receiver operating characteristic (ROC) curve analysis. In addition, we performed KM and ROC curve analyses on the validation cohort GSE17539. Finally, we investigated the performance of hub genes in predicting prognosis using KM curves and compared their expression levels in tumor and normal tissues.

### Comprehensive analysis and functional enrichment analyses

A univariate or multivariate Cox regression analysis of risk scores, age, M–N–T stages, gender, and stage was performed to identify whether risk scores predict prognosis independently. Constructing a nomogram was used for predicting COAD patient prognosis, combining risk scores and several clinical factors. An estimation of nomogram accuracy was made using the calibration curve. The risk group clinical characteristics were compared, including survival status, stage, and M–N–T stages. Also, enrichment pathway analyses were performed via gene sets obtained from Gene Ontology (GO), Kyoto Encyclopedia of Genes and Genomes (KEGG), and Hallmark gene sets.

### Genomic alterations and immune profiling of HDAC-related features

We downloaded somatic mutation data and copy number variation (CNV) data by accessing the TCGA database. Then, we calculated the tumor mutational load (TMB) of each patient and performed a risk group analysis incorporating different TMB levels to assess the overall survival (OS) of the patients. To assess the distribution of CNVs, we employed the genomic identification (GISTIC) algorithm to estimate CNVs at important targets. In addition, we evaluated immune cells and functional scores in the tumor microenvironment using the single-sample genomic enrichment analysis (ssGSEA) algorithm. Subsequently, we compared the expression of 50 immune checkpoints between different risk groups. After considering the clinical significance of antibodies against PD-1/-L1/-L2 and CTLA4, we further explored the relationship between risk scores and these immune checkpoints.

### Therapy response prediction

First, the immunotherapy response for each COAD patient was assessed relying on the gene expression patterns through the tumor immune dysfunction and exclusion (TIDE) (http://tide.dfci.harvard.edu). Immunotherapy is more effective, to a great extent, for patients with lower TIDE scores^13^. Furthermore, the Submap algorithm was used to estimate COAD patient response to the treatment of anti-PD1 and -CTLA4. The Genomics of Drug Sensitivity in Cancer (GDSC) data enabled predicting patient sensitivity to chemotherapeutic drugs, apart from immunotherapy.

### Cell culture and transfection

The HCT116 and HT29 human colon cancer cell lines were purchased from the Cell Bank of the Chinese Academy of Sciences (Beijing, China), and were cultured in Dulbecco’s modified Eagle medium (DMEM) (Gibco, Grand Island, NY) supplemented with 10% foetal bovine serum (FBS) at 37 °C. HCT116 and HT29 cells were inoculated in 6-well plates at a density of 4 × 10^5^/mL for 24 h. According to the reagent instructions, synthesized small interfering RNA (siRNA) was transfected into cells by Lipofectamine 3000 (Invitrogen, Carlsbad, CA, USA). After 24 h of transfection, the silencing efficiency was tested with quantitative reverse transcription polymerase chain reaction (qRT-PCR). All siRNA sequences for BRD3 are available in Table S1.

### RNA extraction and RT-PCR

The total RNA was extracted from HCT116 and HT29 cell lines using TRIzol Reagent (Invitrogen). Then, isolated RNA was reverse transcribed via High Capacity cDNA Reverse Transcription kits (Vazyme, China). Finally, qRT-PCR was processed with SYBR Green PCR Master Mix (Vazyme, China). GAPDH was applied as an internal control. The primers of BRD3 are shown in Table S2.

### Proliferation, migration, and apoptosis experiments

COAD cells of different groups were seeded into 96-well plates with serum-free medium, and 10 μL of Cell Counting Kit-8 (CCK-8) reagent was added after incubation for 24 h, 48 h, and 72 h. Then, the cells were cultured in the dark for 1–4 h. The absorbance at 450 nm was measured via an automatic microplate reader (Synergy4; BioTek, USA). Moreover, COAD cell lines were inoculated into a 6-well plate and when cell fusion approached 90%, the 1 mL pipette tip was used to scratch. After being treated with 1 μg/mL mitomycin C in a serum-free medium for 24 h, the width of the wounds was examined under a microscope (ICX41, SDPTOP, China) (magnification, 40×). The wounds were photographed at 0 h and 24 h after scratching, and the ImageJ software was applied to measure the wound widths and wound closure rates. A transwell migration experiment was also applied to examine cellular migration ability with a 6.5-mm diameter Transwell chamber (Corning, USA). HCT116 and HT29 cells (5 × 10^5^) in 200 μL serum-free DMEM were seeded into the upper chamber in the Transwell chamber, and 700 μL DMEM with 10% FBS was added into the lower chamber. After incubating at 37 °C for 24 h, the membranes of the chamber were fixed with 4% paraformaldehyde for 30 min. Then, 0.1% crystal violet was utilized to stain the migrated cells for 30 min at 37 °C, and the cell number was counted by an inverted biological microscope (ICX41, SDPTOP, China) (magnification, 200×). Five areas were randomly chosen in each group. All experiments were performed independently three times. Data are shown as the mean ± standard deviation (SD). Transfected COAD cells of different groups were seeded in 6-well plates. Then approximately 1 × 10^5^ cells, including suspended cells, were harvested by ethylene diamine-tetra acetic acid (EDTA)-free trypsinization and washed twice with cold phosphate buffer saline (PBS). According to the instruction of the Annexin V-fluorescein isothiocyanate (FITC)/propidium iodide (PI) apoptosis kit (MultiSciences, China), cell apoptosis was detected by a CytoFLEX-3 flow cytometer (BD, USA). Acquired data were analysed via FlowJo software. Three replicates were set in each group, and all experiments were performed independently 3 times.

## Results

### HDAC-related genes are associated with the prognosis of COAD

**Figure 1:**
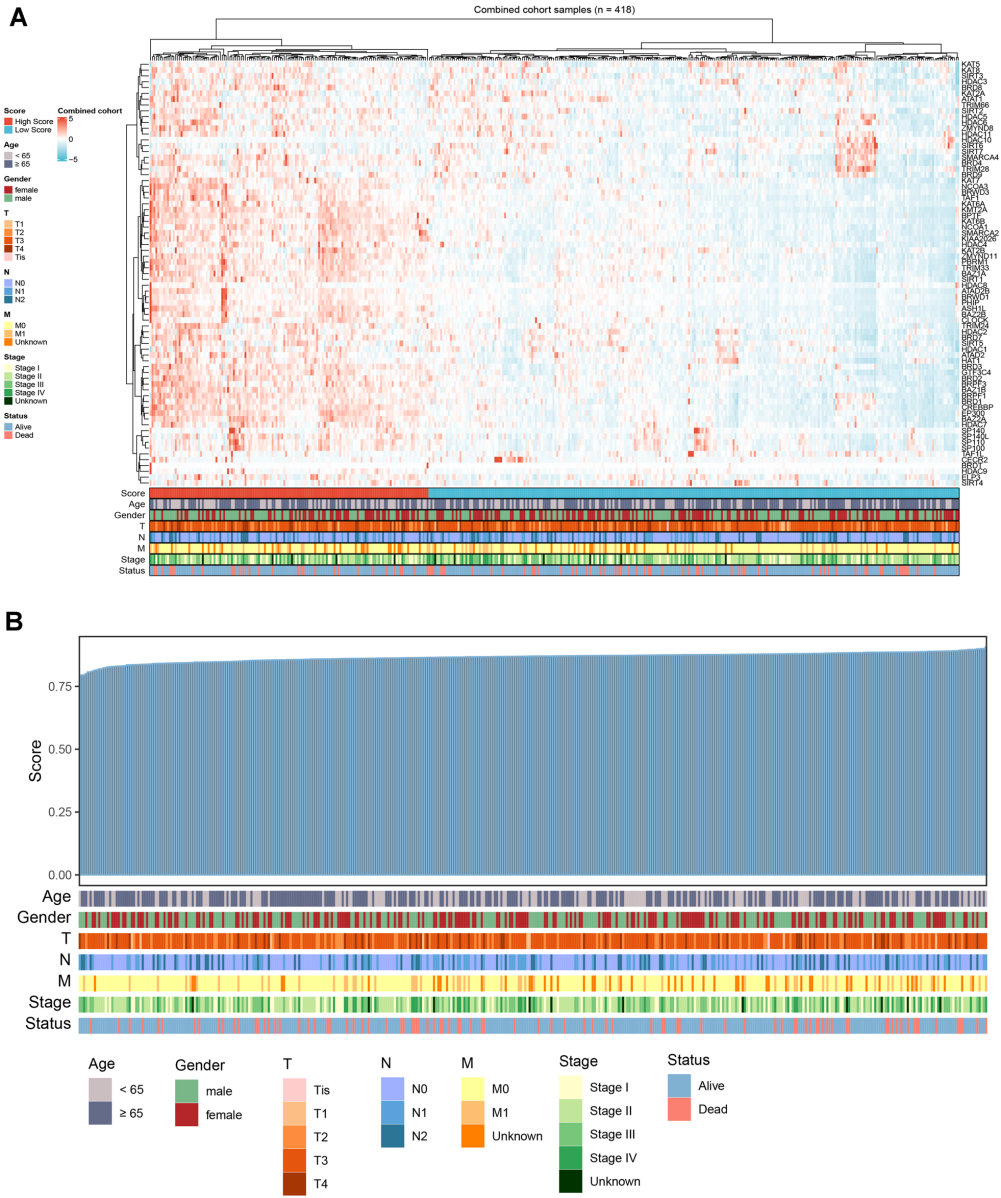
Expression and qualification of HDAC-related genes. *Notes*: (**A**) A heatmap displaying HDAC-related gene expression levels and different clinical characteristics. (**B**) HDAC scores were qualified for each individual and shown with various clinical features.

The expression profiles of HDAC-related genes (KAT, SIRT3, HDAC3, etc.) in 418 COAD patients were displayed as heatmaps. Age, sex, stage, and survival information for each gene were also compared to assess the correlation between HDAC-related genes and COAD (Fig. 1A). The expression levels of HDAC-related genes were shown in Table S3. Patients were then ranked according to HDAC scores and clinical characteristics were listed, and the results showed that HDAC genes were strongly associated with COAD patients, independent of gender and age, and that clinical stage M0 was highly correlated with HDAC expression (Fig. 1B).

**Figure 2:**
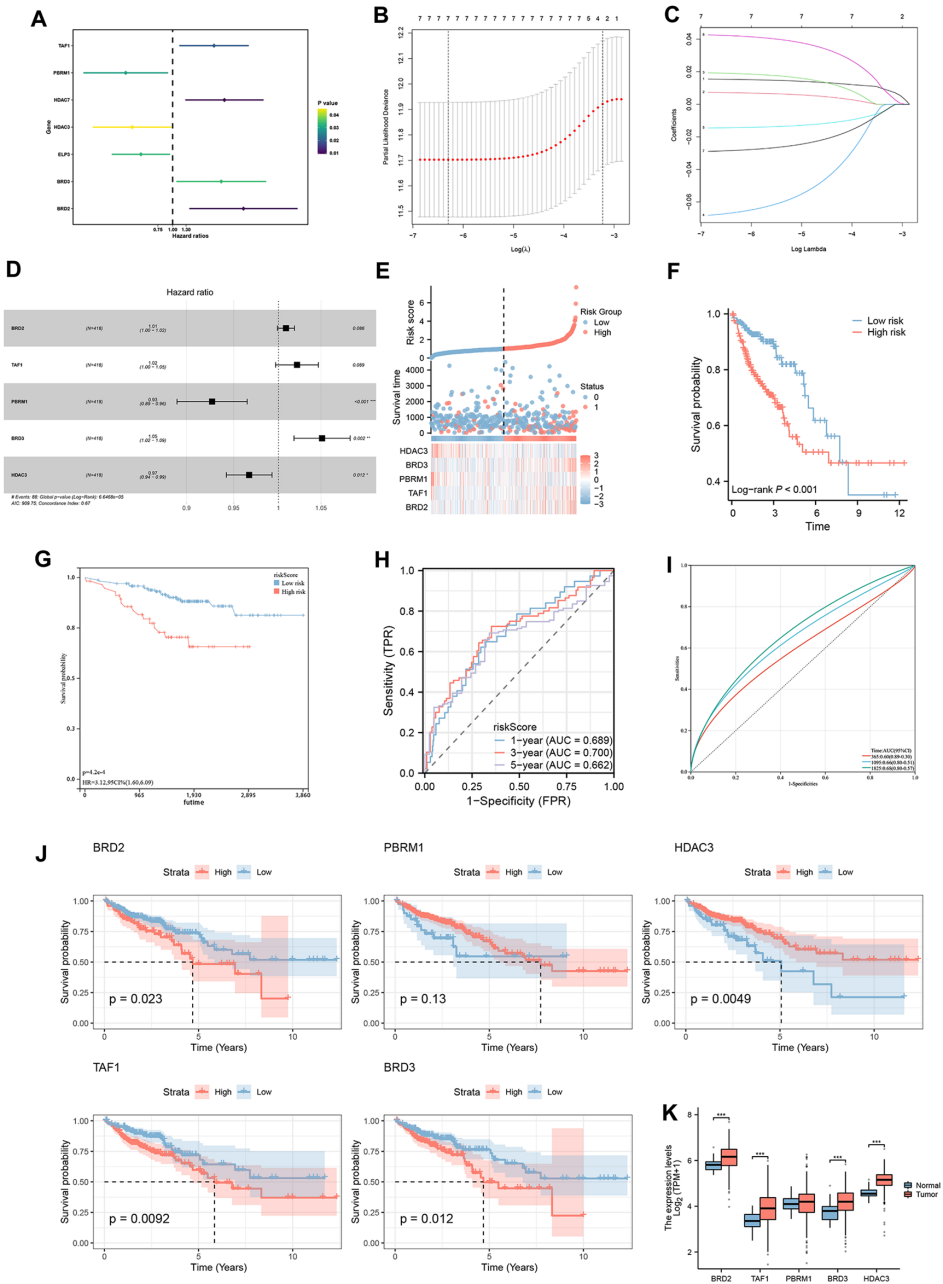
Construction of HDAC-related signature. *Notes*: (**A**) Forest plots reflected the prognostic genes. (**B**, **C**) Cvfit and lambda curves displaying both LASSO regression analysis and coefficient profiles of the 7 genes related to HDAC. (**D**) Forest plot for the multivariate analysis of hub genes. (**E**) An analysis of risk scores, survival status, and hub gene expression distribution. (**F**) Survival curve of TCGA and (**G**) GEO cohorts. (**H**) ROC Curve Analysis of the Risk Score for TCGA Dataset. The graph illustrates the predictive performance of the risk score over different timeframes. (**I**) ROC Curve Analysis of the Risk Score for GEO Dataset. (**J**) Survival curves of hub genes. (**K**) Comparing hub gene expressions between tumors and normal tissues. *p < 0.05, **p < 0.01, ***p < 0.001.

To investigate the prognostic relationship between HDAC-related genes and COAD, univariate Cox analysis was used to identify prognostic genes based on the expression levels of HDAC-related genes (Fig. 2A), and the results showed that the predictors of TAF1, HDAC7, and BRD3 were associated with poorer survival. After reducing the number of genes by LASSO analysis, a prognostic risk assessment model was established using multivariate Cox analysis (Fig. 2B–D). Results showed the relationship between the expression levels of the five pivotal genes (BRD2, TAF1, PBRM1, HDAC3, BRD3) and the risk score. In addition, patients with a high-risk score were more likely to die, and survival was not as good as patients with a low-risk score (Fig. 2E, F). The GSE17538 cohort validated using KM curves yielded the same results (Fig. 2G). The ROC curves showed an area under the curve (AUC) values of 0.689, 0.700, and 0.662 for predicting 1-, 3-, and 5-year survival, respectively (Fig. 2H). The 1-, 3- and 5-year AUCs for the validation cohort were 0.600, 0.660, and 0.680, respectively (Fig. 2I). In addition, the AUC of the ROC curve for risk scoring of diagnostic tumors and normal tissues was 0.705 (Figure S1A). In addition, we found that BRD2, HDAC3, TAF1, and BRD3 were significant predictors of outcome (Fig. 2J, K). The above results indicated that HDAC-related genes were closely related to COAD prognosis, suggesting that HDAC-related genes might be targets for COAD prognosis.

### Analyses of clinical characteristics and function enrichment

**Figure 3:**
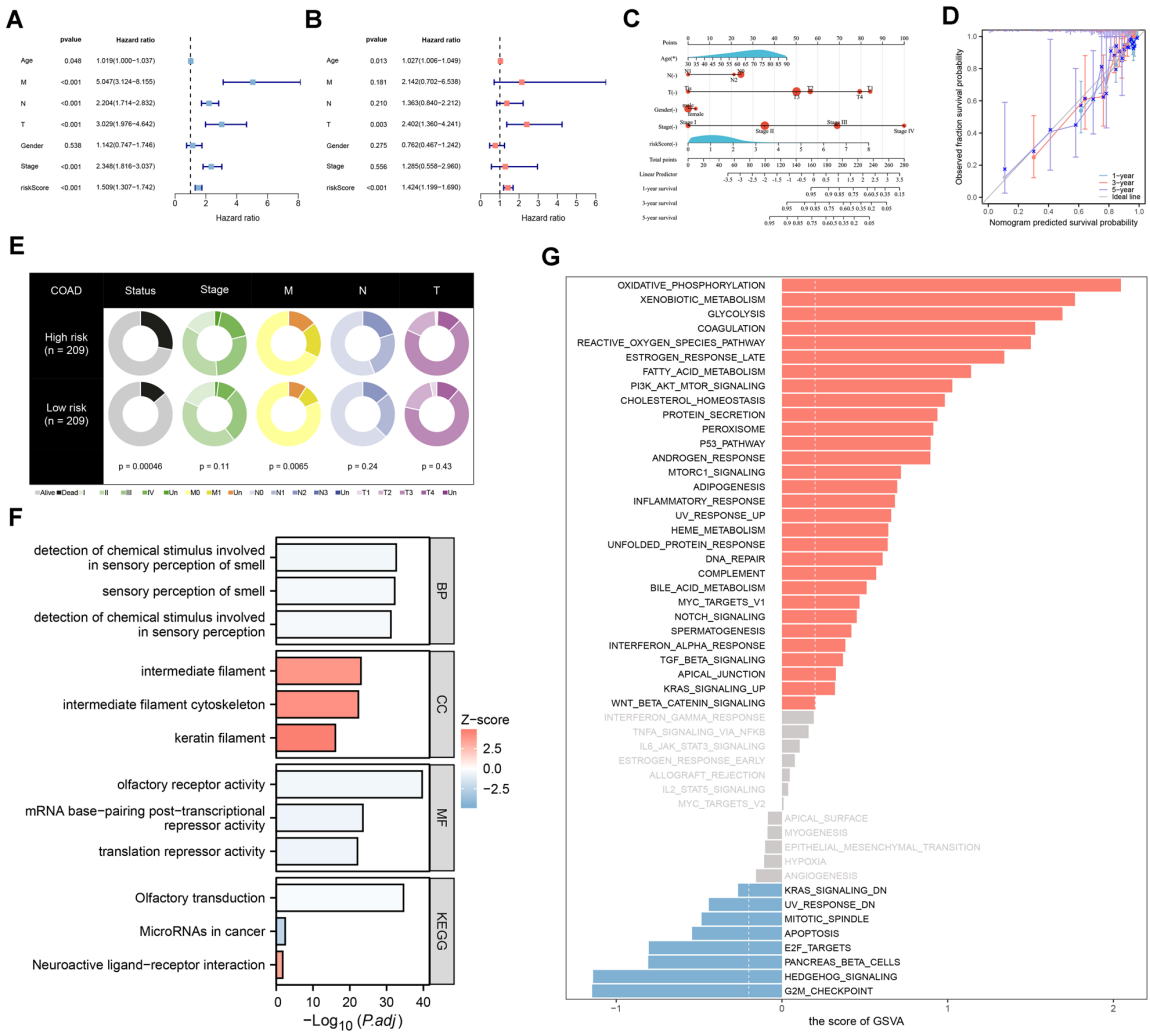
Analyzing the signature and functional enrichment. *Notes*: (**A**, **B**) The univariate and multivariate analyses of the survival time of signature. (**C**) Prediction of 1-, 3-, and 5-year survival probabilities relying on a nomogram. (**D**) Calibration curve used to estimate nomogram accuracy. (**E**) Pie charts displaying the risk group clinical characteristics. (**F**) Displaying enriched biological processes using GO and KEGG. (**G**) Identifying significant enrichment pathways among the risk groups. *p* < 0.05.

Based on univariate and multivariate Cox analyses, the risk score was identified as an independent predictor of prognosis (Fig. 3A, B). To predict OS in patients with COAD, we constructed a nomogram including age, N–T stage, gender, stage, and risk score (Fig. 3C), and validated the accuracy of the nomogram in predicting survival using calibrated plots (Fig. 3D). By differentially analyzing the clinical characteristics, we found that patients in the high-risk group were more likely to die and develop metastases (Fig. 3E).

**Figure 4:**
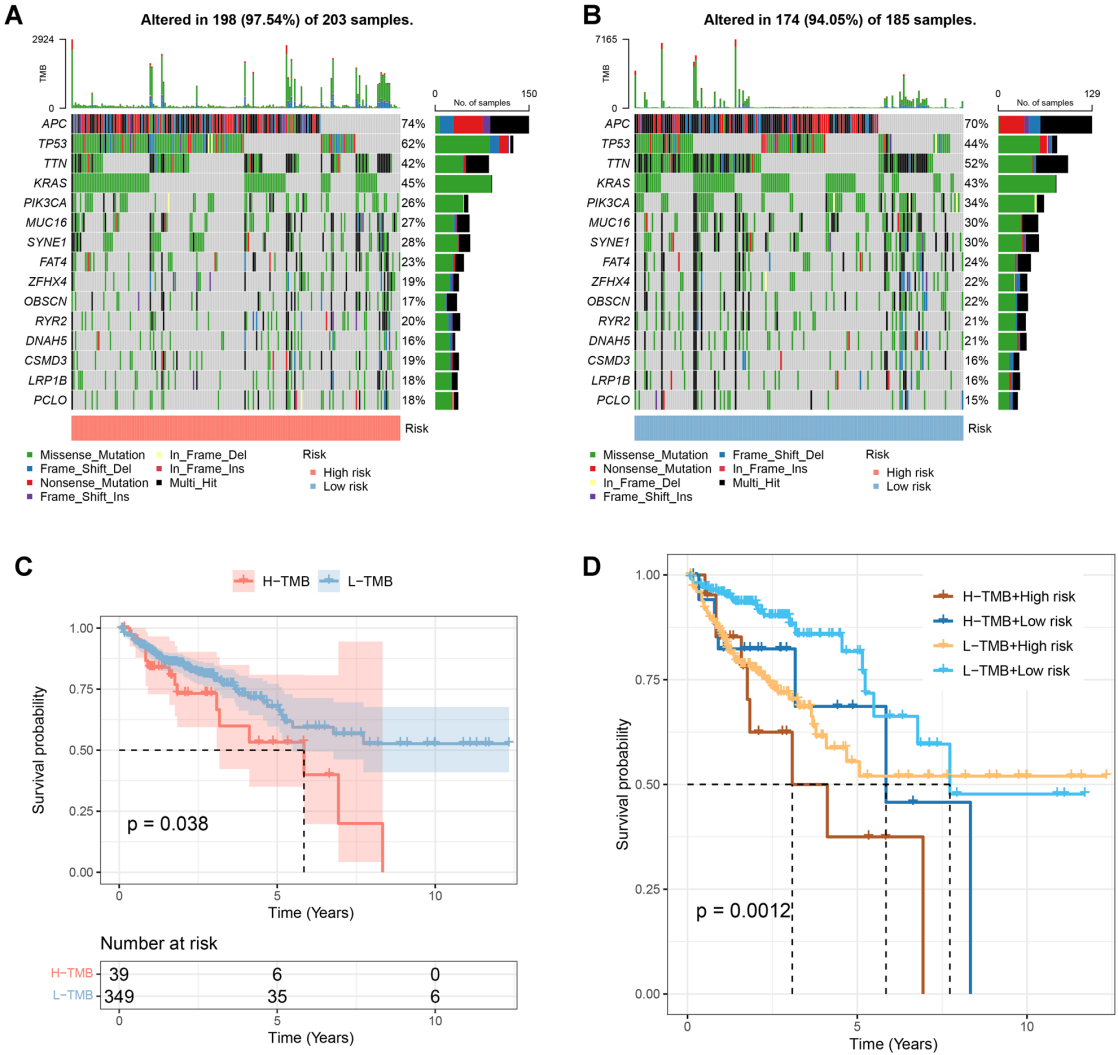
Genomic mutation analysis for HDAC-related signature. *Notes*: (**A**, **B**) The gene mutation frequency in the risk groups. (**C**) A lower TMB (mut/Mb) level was correlated to better patient outcomes. (**D**) A low TMB (mut/Mb) level in a low-risk group led to the best outcomes.

Further functional enrichment analysis was performed to explore the functions associated with HDAC-related features. The top 12 functions in terms of GO and KEGG enrichment were detection of chemical stimuli involved in olfactory perception, olfactory perception, detection of chemical stimuli involved in olfactory perception, intermediate filaments, cytoskeleton of intermediate filaments, keratin filaments, olfactory receptor activity, mRNA base-pairing post-transcriptional repression activity, translational repression activity, olfactory transduction, microRNA in cancer, and neuroactive ligand-receptor interactions (Fig. 3F). In the high-risk group, the top five pathways enriched in the marker gene set were oxidative phosphorylation, xenobiotic metabolism, glycolysis, coagulation, and reactive oxygen species pathways. In the low-risk group, the top five functions enriched in the marker gene set were G2M checkpoint, hedgehog signaling, pancreatic β-cells, E2F targets, and apoptosis (Fig. 3G). APC, TP53, TTN, KRAS, PIK3CA, MUC16, SYNE1, FAT4, ZFHX4, OBSCN, RYR2, DNAH5, CSMD3, LRP1B, and PCLO are more frequently mutated among patients with both TMB levels (Fig. 4A, B).

According to the survival curves, COAD patients with lower TMB levels had higher survival rates (Fig. 4C). We further analyzed their impact on prognosis by combining TMB levels and risk groups. The results showed that low-risk patients with lower TMB levels had the highest survival rate (Fig. 4D).

**Figure 5:**
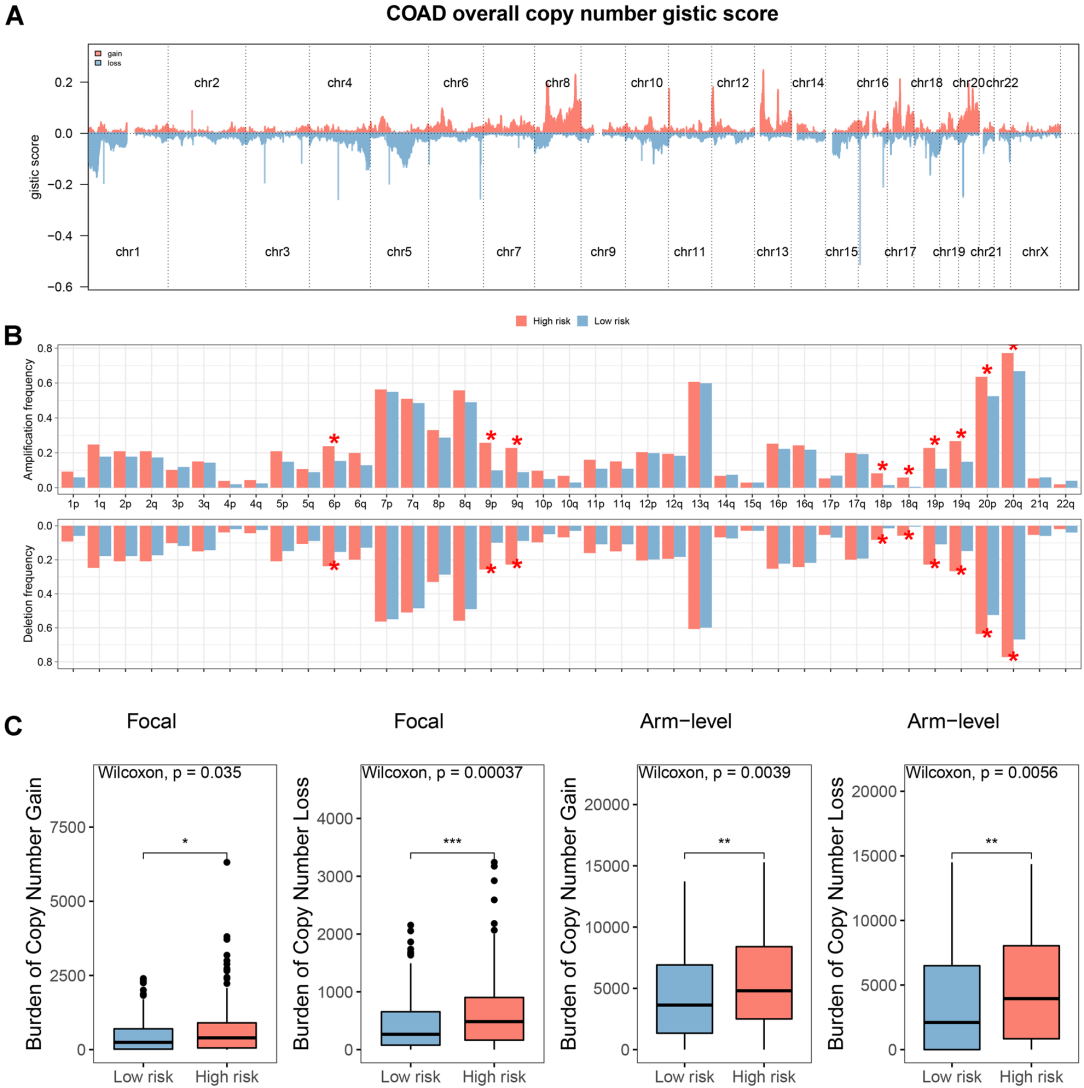
Gene amplification and deletion analyses. *Notes*: (**A**) A summary of the copy number scores was shown. (**B**) A comparison between the risk groups in copy number amplifications and deletions. (**C**) At focal and arm levels, comparing the burden of copy number gain and loss between the risk groups. *p < 0.05, **p < 0.01, ***p < 0.001.

**Figure 6:**
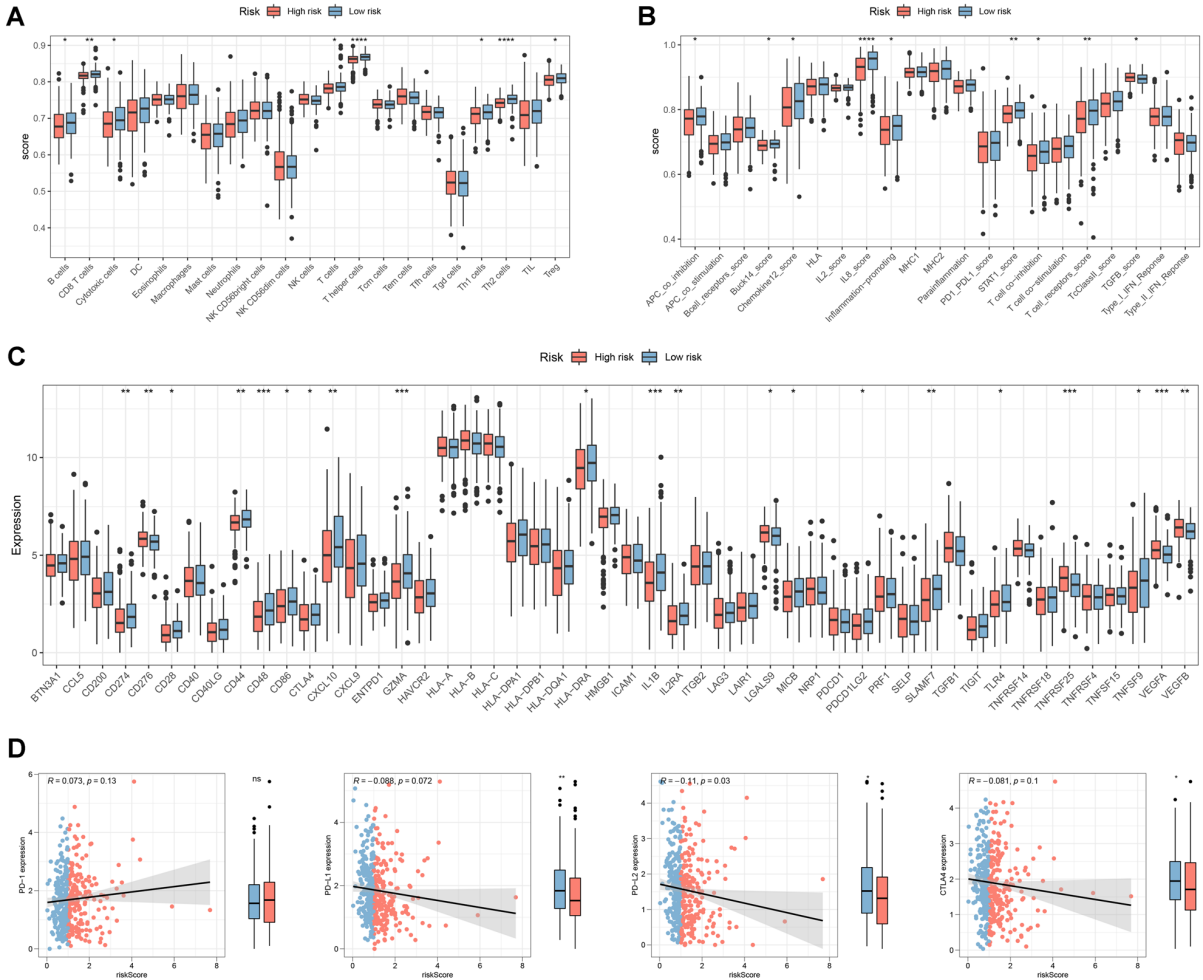
Analyses of immune infiltration in COAD patients. *Notes*: (**A**) Compares the differences in abundance or activity of various types of immune cells between high-risk and low-risk groups. Each box plot represents a specific type of immune cell (such as B cells, CD8 + T cells, macrophages, etc.). (**B**) Compares the differences between the two groups in multiple immune-related functional scores. These scores reflect indicators of various immune inhibitory or activating states in the tumor microenvironment. (**C**) Immune checkpoint expressions were compared between the risk groups. (**D**) Analysis of the correlations between risk scores and PD-1/-L1/-L2 and CTLA4 expressions. *p < 0.05, **p < 0.01, ***p < 0.001, ****p < 0.0001.

We used the GISTIC algorithm to estimate the profiles of the CNV characteristics between the risk groups, and the GISTIC scores for the total number of copies are shown in Fig. 5A. Significant differences in amplification and deletion frequencies were observed between risk groups (Fig. 5B). In addition, patients in the high-risk group had a higher copy number loss and gain burden at the lesion and arm level (Fig. 5C). By assessing immune cells and function in the risk group, we found that patients in the low-risk group had increased numbers of B cells, CD8 + T cells, cytotoxic cells, and T cells (Fig. 6A). In addition, T-cell receptor and TGF-β levels were also increased, suggesting a better anti-tumor effect in patients in the low-risk group (Fig. 6B). The expression of immune checkpoints also differed between the risk groups, with higher expression in the low-risk group, indicating a better response to immunotherapy (Fig. 6C). The expression of PD-L1/-L2 and CTLA4 was negatively correlated with the risk score (Fig. 6D).

**Figure 7:**
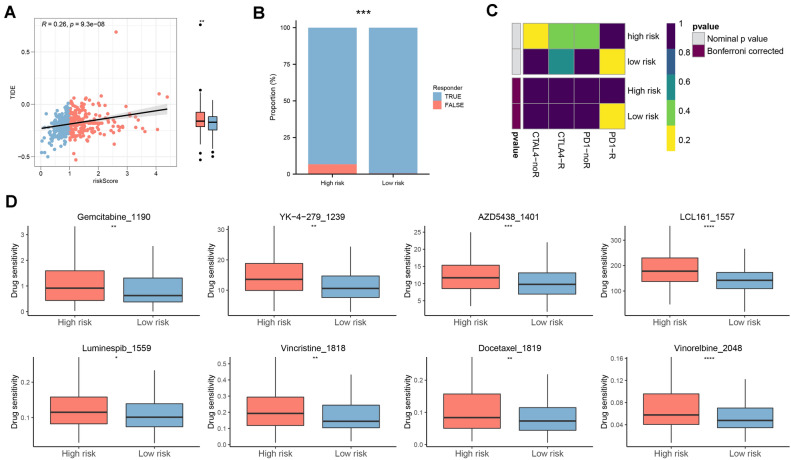
Prediction of immuno- and chemo-therapy efficacy in COAD patients. *Notes*: (**A**) The difference in TIDE between the risk groups. (**B**) Comparison of immunotherapy response numbers between the two risk patients. (**C**) Submap analysis for HDAC-related signature in COAD. (**D**) Box plots of estimated 8 chemotherapeutic agent-drug sensitivity in the risk groups. *p < 0.05, **p < 0.01, ***p < 0.001, ****p < 0.0001.

The TIDE algorithm showed positive risk values relative to TIDE values, with the lower risk group having lower values (Fig. 7A) and a greater response to immunotherapy (Fig. 7B). Previous analyses showed that the CD8^+^ T-cell infiltration score and the TMB score were higher in the low-risk group than in the high-risk group. Combined with the submapping analysis, patients in the low-risk group may benefit from anti-PD-1 therapy (Fig. 7C). Additionally, we determined the chemosensitivity of high-risk patients to eight chemotherapeutic agents (gemcitabine, YK-4-279, AZD5438, LCL161, luminespib, vincristine, docetaxel, and vincristine) (Fig. 7D). Taken together, we identified the expression level of HDAC-related genes as one of the important factors in assessing the survival and prognosis of COAD patients, suggesting that HDAC may play a role in the development and progression of COAD. In addition, further functional enrichment analysis also revealed that HDAC-related features were associated with biological functions and pathways in COAD. These results suggested that HDAC and its related genes may be involved in the regulation of the pathogenesis of COAD, which may have an impact on tumor cell survival, proliferation, metastasis, and other biological processes.

### BRD3 increases the proliferation, migration and represses apoptosis in HCT116 and HT29 cells

**Figure 8:**
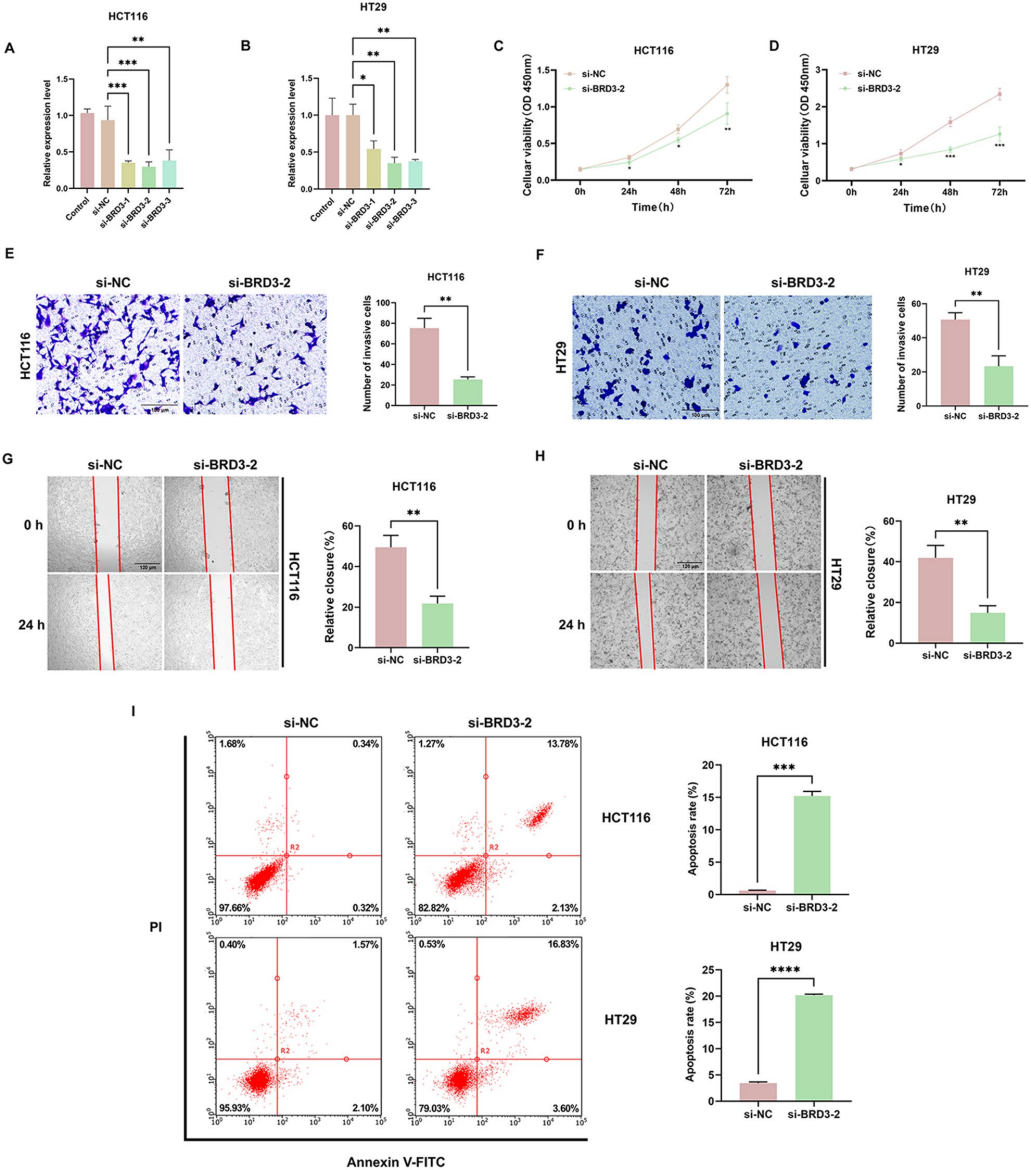
The function of BRD3 on the proliferation, migration, and apoptosis of COAD cells. Notes: The results of qRT-PCR showed that si-BRD3-2 successfully knocked down the expression of BRD3 in HCT116 (**A**) and HT29 (**B**) cells. The knockdown of BRD3 inhibited the proliferation ability in both HCT116 (**C**) and HT29 (**D**) cells compared with the si-NC group. The depletion of BRD3 significantly repressed the migration of HCT116 (**E**, **G**) and HT29 (**F**, **H**) cells. (**I**) Flow cytometry showed that BRD3 knockdown could promote the apoptosis in HCT116 and HT29 cells. *p < 0.05, **p < 0.01, ***p < 0.001, *p*-value based on t-test. All experiments were implemented separately in triplicate.

To further explore the function of BRD3 in COAD cells, we investigated the impact of the BRD3 gene on COAD proliferation, migration, and apoptosis. si-BRD3-2 successfully knocked down the expression of BRD3 in HCT116 and HT29 cells (Figs. 8A, B). CCK-8 analysis showed that BRD3 knockdown inhibited the proliferation ability of both HCT116 and HT29 cells compared with the si-NC group (Figs. 8C, D). In addition, the transwell migration assay and wound healing assay revealed that the depletion of BRD3 significantly repressed the migration of HCT116 and HT29 cells (Figs. 8E, H). The flow cytometry results demonstrated that BRD3 significantly suppressed COAD apoptosis (Fig. 8I).

## Discussion

As a result of its high morbidity and mortality, COAD has become a threat to public health on a global scale^14^. Despite lifestyle modifications and screening tools that have improved, COAD is still increasing in incidence and mortality, and most tumors have aggressive pathology^3^. The poor prognosis of COAD has been improved with new therapeutic strategies targeting specific genomic alterations such as anti-EGFR and anti-VEGFR antibodies^15^. Additionally, immune-checkpoint inhibitors (ICIs) can prolong patients’ OS, but some patients may be resistant to ICIs. As COAD is characterized by high genetic heterogeneity, we should develop optimal models to predict patient responses to immunotherapy or further understand their genetic mutations^16^.

The HDAC families regulate gene transcription and are crucial in several biological processes; moreover, abnormal HDACs are involved in cancer development and metastasis. Inhibiting HDACs may control the expression of genes that control the cell cycle and induce apoptosis through the alternation of their gene expressions. By regulating the expression of proapoptotic BCL2 family proteins, including Bim and Bmf, HDAC inhibitors can mediate cell apoptosis^17^. In order to effectively treat COAD, it is important to explore the different impacts of HDACs after regulating COAD gene expression and to investigate novel therapeutic agents targeting HDACs^11^.

Herein, bioinformatics methods were used for constructing an innovative HDACs-related prognostic signature, which consists of five genes (BRD2, PBRM1, HDAC3, TAF1, and BRD3) was used to divide COAD patients with different outcomes. In tumor tissues and normal tissues, four hub genes were differentially expressed. Additionally, prostate cancer tissues had significantly higher BRD2 and BRD3 expression levels than normal prostate tissues^18^. The BRD2 expression level exhibited a positive correlation to the mortality rate of prostate cancer patients^19^. By silencing BRD3 with BRD3-specific siRNA, lung cancer cells are inhibited from proliferating and migrating, and their apoptosis is promoted^20^. The PBRM1 gene is crucial in suppressing non-small cell lung cancer (NSCLC) tumor function, and its loss may lead to renal cell carcinoma tumor growth^21,22^. As one of the most important HDACs for regulating the cell cycle, HDAC3 participated in developing liver cancer in mice; when HDAC3 is deleted can result in a significant loss of genomic stability as well as tumorigenesis^23^. Further, TAF1 expression was increased in NSCLC, and it regulates apoptosis-related gene expression and cell cycle regulation as well^24,25^.

Using univariate and multivariate independent prognostic analyses verified that the signature is a crucial independent OS predictor, demonstrating the signature prognostic value using external validation. Consequently, the characteristics of genomic alteration associated with the constructed prognostic HDAC-related signature model were evaluated. The molecular alterations of COAD control the functions of numerous signaling pathways and contribute to its heterogeneity^26^. There is a crucial role for tumor stability at various genetic aspects in the sophisticated tumor molecular biology as well as how tumors respond to immunotherapy and chemotherapy^27^. We calculated somatic mutations for every patient and identified the top 15 genes among the risk groups. Most COAD patients had mutated APC genes, which was the first step to tumor formation when the normal colorectal epithelium transformed into carcinoma^28^. KRAS overexpression has been correlated to tumor metastasis and poor prognosis^29^. TMB levels in colorectal cancer patients have a significant impact on survival. We observed a higher survival rate in patients with lower TMB levels, which may be related to the association of TMB levels with tumor immunogenicity and tumor microenvironment. In addition, by combining TMB levels with risk groups, we were able to more accurately predict the prognosis of colorectal cancer patients; low-risk patients with lower TMB levels had the highest survival rates, suggesting that TMB levels and risk scores are important prognostic indicators. In addition to TMB levels, we also found significant differences in the frequency of copy number amplifications or deletions among patients in the risk groups, suggesting that genetic characteristics of tumors are closely related to prognosis. When we further analyzed the immune infiltration between the risk groups, we found that the risk score was closely associated with B cells and T cells. Intensive infiltration of B and T cells was associated with a better prognosis, which is consistent with the results of previous studies. Of particular note, there was also a correlation between the risk score and sensitivity to immunotherapy. In the low-risk group, PD-L1/-L2 and CTLA4 were overexpressed, suggesting that immunotherapy may be more effective in these patients. Using subgroup analysis, we also found that anti-PD1 therapy was more likely to be effective in the low-risk group. In addition, risk scores were associated with chemotherapy sensitivity, including sensitivity to chemotherapeutic agents such as gemcitabine, YK-4–279, AZD5438, and docetaxel. These results highlight the importance of risk score as a prognostic factor and its association with immunotherapy and chemotherapy sensitivity, providing useful information for individualized treatment of colorectal cancer patients^30^. The potential value of HDAC-associated gene signatures lies in the additional molecular information they provide about patients with COAD, contributing to a more comprehensive understanding of the biology and disease progression of COAD patients. By integrating these gene signatures into existing diagnostic models, we can more accurately assess patient risk and prognosis. For example, HDAC-associated gene signatures may be associated with key factors such as tumor grade, risk of metastasis, and response to treatment in COAD. By taking this information into account, physicians can better develop individualized treatment plans to improve treatment outcomes and survival rates. Specifically, these genetic features may include expression levels of HDAC family members, information on HDAC-associated mutations, and biomarkers related to the HDAC pathway. By comprehensively analyzing these features, the molecular typing of patients can be identified and their response to different treatment strategies can be predicted. In addition, these genetic features may also provide an important reference for the prognostic assessment of COAD patients and help differentiate between patient survival and disease progression. Overall, the integration of HDAC-associated gene signatures into a diagnostic model could provide a more comprehensive and accurate patient assessment and more targeted guidance for treatment decisions in COAD patients. This comprehensive diagnosis. Fourth, bioinformatics analysis, while insightful, inherently lacks the capacity to fully capture the complexities of biological systems in vivo, offering more indicative insights rather than exact representations. It is essential to interpret these computational findings with caution. In conclusion, the prognostic HDAC-related signature requires further validation and explanation.

## Conclusion

The practical significance and future research direction of this study are crucial. First, by identifying HDAC-related genes as an important indicator of COAD prognosis, we can not only more accurately assess the survival risk of patients, but also provide more reliable guidance for personalized treatment plans. Second, this study provides an in-depth exploration of the biological mechanisms of COAD development, revealing the importance of HDAC-related genes in the pathogenesis of COAD and providing a new perspective for further understanding tumor development. In the future, the sample size can be further expanded to verify the reliability of the findings and explore the potential therapeutic targets of HDAC-related genes to develop more effective therapeutic strategies. This study provides an important foundation for the prognostic assessment and treatment of COAD and makes a positive contribution to the development of the field.

### Supplementary Information

Supplementary Legends.

Supplementary Figure S1.

Supplementary Table S1.

Supplementary Table S2.

Supplementary Table S3.

## Data Availability

The datasets provided for this study can be found in online repositories. The RNA expression profiles, mutations, and clinical data of COAD can be downloaded from the TCGA database (https://portal.gdc.cancer.gov). The GSE17538 and GSE17539 cohort can be downloaded from the GEO database (https://www.ncbi.nlm.nih.gov).

## Abbreviations

HDAC: Histone deacetylase
COAD: Colon adenocarcinoma
HAT: Histone acetyltransferase
HDACi: Inhibitors of HDAC
KM: Kaplan–Meier
OS: Overall survival
ROC: Receiver operating characteristic
GO: Gene Ontology
KEGG: Kyoto Encyclopedia of Genes and Genomes
CNV: Copy number variation
TMB: Tumor mutation burden
GISTIC: Genomic Identification of Significant Targets in Cancer
ssGSEA: Single-sample gene set enrichment analysis
TIDE: Tumor immune dysfunction and exclusion
GDSC: Genomics of Drug Sensitivity in Cancer
DMEM: Dulbecco’s modified Eagle medium
FBS: Foetal bovine serum
siRNA: Small interfering RNA
qRT-PCR: Quantitative reverse transcription polymerase chain reaction
CCK-8: Cell Counting Kit-8
SD: Standard deviation
EDTA: Ethylene diamine-tetra acetic acid
PBS: Phosphate buffer saline
FITC: Fluorescein isothiocyanate
PI: Propidium iodide
AUC: Area under the curve
ICI: Immune-checkpoint inhibitor
NSCLC: Non-small cell lung cancer

## References

[1] Siegel RL, Miller KD, Fuchs HE, Jemal A (2022). Cancer statistics, 2022. CA Cancer J. Clin..

[2] Obaro AE, Burling DN, Plumb AA (2018). Colon cancer screening with CT colonography: Logistics, cost-effectiveness, efficiency and progress. Br. J. Radiol..

[3] Siegel RL, Miller KD, Goding Sauer A, Fedewa SA, Butterly LF, Anderson JC (2020). Colorectal cancer statistics, 2020. CA Cancer J. Clin..

[4] Miller KD, Nogueira L, Mariotto AB, Rowland JH, Yabroff KR, Alfano CM (2019). Cancer treatment and survivorship statistics 2019. CA Cancer J. Clin..

[5] Tarazona N, Gimeno-Valiente F, Gambardella V, Huerta M, Roselló S, Zuniga S (2020). Detection of postoperative plasma circulating tumour DNA and lack of CDX2 expression as markers of recurrence in patients with localised colon cancer. ESMO Open..

[6] Tarazona N, Gimeno-Valiente F, Gambardella V, Zuñiga S, Rentero-Garrido P, Huerta M (2019). Targeted next-generation sequencing of circulating-tumor DNA for tracking minimal residual disease in localized colon cancer. Ann. Oncol..

[7] Herrera M, Berral-González A, López-Cade I, Galindo-Pumariño C, Bueno-Fortes S, Martín-Merino M (2021). Cancer-associated fibroblast-derived gene signatures determine prognosis in colon cancer patients. Mol. Cancer.

[8] Wu Z, Lu Z, Li L, Ma M, Long F, Wu R (2022). Identification and validation of ferroptosis-related LncRNA signatures as a novel prognostic model for colon cancer. Front. Immunol..

[9] Bosch-Presegué L, Vaquero A (2015). Sirtuin-dependent epigenetic regulation in the maintenance of genome integrity. FEBS J..

[10] Mertsch S, Krämer OH (2017). The interplay between histone deacetylases and rho kinases is important for cancer and neurodegeneration. Cytokine Growth Factor Rev..

[11] Gallinari P, Di Marco S, Jones P, Pallaoro M, Steinkühler C (2007). HDACs, histone deacetylation and gene transcription: from molecular biology to cancer therapeutics. Cell Res..

[12] Zhu P, Martin E, Mengwasser J, Schlag P, Janssen KP, Göttlicher M (2004). Induction of HDAC2 expression upon loss of APC in colorectal tumorigenesis. Cancer Cell.

[13] Jiang P, Gu S, Pan D, Fu J, Sahu A, Hu X (2018). Signatures of T cell dysfunction and exclusion predict cancer immunotherapy response. Nat. Med..

[14] Kocarnik JM, Compton K, Dean FE, Fu W, Gaw BL, Harvey JD (2022). Cancer incidence, mortality, years of life lost, years lived with disability, and disability-adjusted life years for 29 cancer groups from 2010 to 2019: A systematic analysis for the global burden of disease study 2019. JAMA Oncol..

[15] Bekaii-Saab T, Kim R, Kim TW, O'Connor JM, Strickler JH, Malka D (2019). Third- or later-line therapy for metastatic colorectal cancer: Reviewing best practice. Clin. Colorectal Cancer.

[16] Morse MA, Hochster H, Benson A (2020). Perspectives on treatment of metastatic colorectal cancer with immune checkpoint inhibitor therapy. The Oncologist.

[17] Bolden JE, Peart MJ, Johnstone RW (2006). Anticancer activities of histone deacetylase inhibitors. Nat. Rev. Drug Discov..

[18] Wang C, Leavenworth J, Zhang C, Liu Z, Yuan KY, Wang Y (2022). Epigenetic regulation of EIF4A1 through DNA methylation and an oncogenic role of eIF4A1 through BRD2 signaling in prostate cancer. Oncogene.

[19] Urbanucci A, Barfeld SJ, Kytölä V, Itkonen HM, Coleman IM, Vodák D (2017). Androgen receptor deregulation drives bromodomain-mediated chromatin alterations in prostate cancer. Cell Rep..

[20] Lee JH, Yoo SS, Hong MJ, Choi JE, Kang HG, Do SK (2022). Epigenetic readers and lung cancer: The rs2427964C>T variant of the bromodomain and extraterminal domain gene BRD3 is associated with poorer survival outcome in NSCLC. Mol. Oncol..

[21] Zhou H, Liu J, Zhang Y, Huang Y, Shen J, Yang Y (2020). PBRM1 mutation and preliminary response to immune checkpoint blockade treatment in non-small cell lung cancer. NPJ Precis. Oncol..

[22] Pawłowski R, Mühl SM, Sulser T, Krek W, Moch H, Schraml P (2013). Loss of PBRM1 expression is associated with renal cell carcinoma progression. Int. J. Cancer.

[23] Bhaskara S, Knutson SK, Jiang G, Chandrasekharan MB, Wilson AJ, Zheng S (2010). Hdac3 is essential for the maintenance of chromatin structure and genome stability. Cancer Cell.

[24] Suzuki-Yagawa Y, Guermah M, Roeder RG (1997). The ts13 mutation in the TAF(II)250 subunit (CCG1) of TFIID directly affects transcription of D-type cyclin genes in cells arrested in G1 at the nonpermissive temperature. Mol. Cell. Biol..

[25] Zhang J, Li R, Zhang B, Cui X (2022). TAF1 promotes NSCLC cell epithelial-mesenchymal transition by transcriptionally activating TGFβ1. Biochem. Biophys. Res. Commun..

[26] Vogelstein B, Fearon ER, Hamilton SR, Kern SE, Preisinger AC, Leppert M (1988). Genetic alterations during colorectal-tumor development. N. Engl. J. Med..

[27] Buikhuisen JY, Torang A, Medema JP (2020). Exploring and modelling colon cancer inter-tumour heterogeneity: Opportunities and challenges. Oncogenesis.

[28] Morin PJ (2019). Colorectal cancer: The APC-lncRNA link. J. Clin. Investig..

[29] Huang D, Sun W, Zhou Y, Li P, Chen F, Chen H (2018). Mutations of key driver genes in colorectal cancer progression and metastasis. Cancer Metastasis Rev..

[30] Fridman WH, Zitvogel L, Sautès-Fridman C, Kroemer G (2017). The immune contexture in cancer prognosis and treatment. Nat. Rev. Clin. Oncol..

