# Supplementary material for: Construction and verification of a histone deacetylases-related prognostic signature model for colon cancer
Source: Sci Rep. 2024 Apr 18;14:8983. doi: 10.1038/s41598-024-59724-x (PMC11026370; doi:10.1038/s41598-024-59724-x)
Supplement: Supplementary file 1 — Supplementary Legends. [file 41598_2024_59724_MOESM1_ESM.docx]

**Figure S1 Analysis of immune statuses.** (A) The ROC curve for risk scores. (B-C) The CD8+ T cell infiltration score and TMB score were used to quantify the distinct immune statuses between the high- and low-risk groups.
