# Supplementary figures and images for: Construction and verification of a histone deacetylases-related prognostic signature model for colon cancer
Source: Sci Rep. 2024 Apr 18;14:8983. doi: 10.1038/s41598-024-59724-x (PMC11026370; doi:10.1038/s41598-024-59724-x)

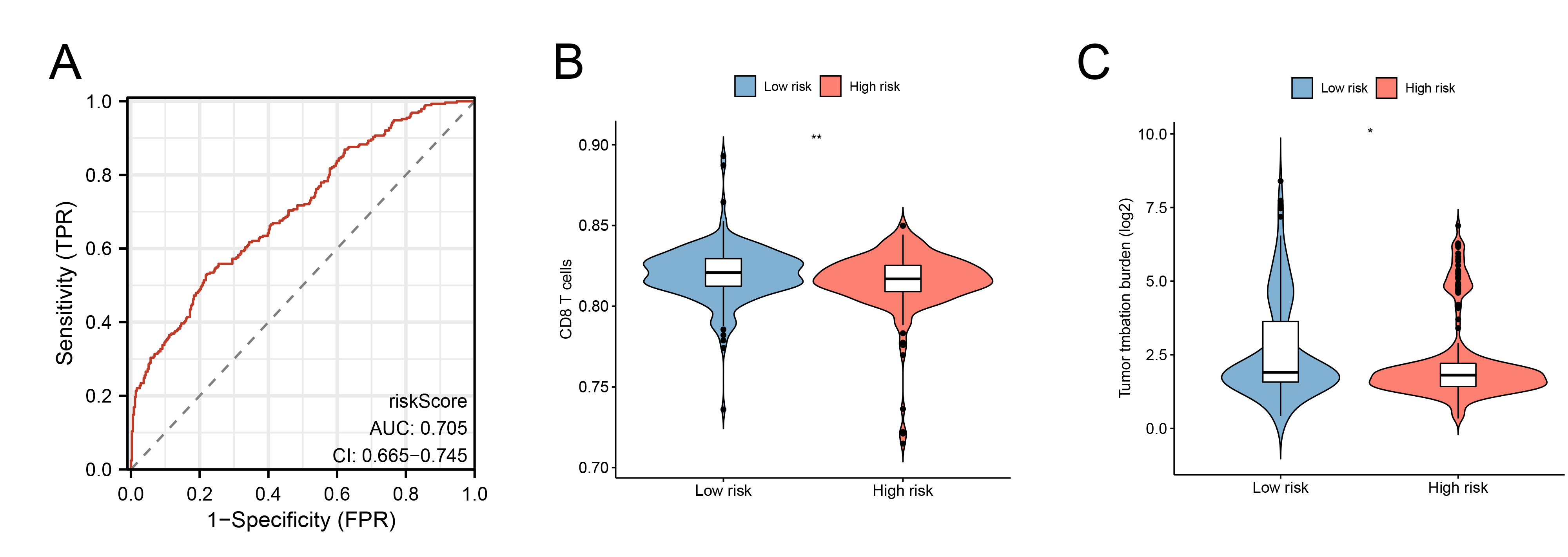

Supplement: Supplementary file 2 — Supplementary Figure S1. [file 41598_2024_59724_MOESM2_ESM.tif]
