# Supplementary material for: Construction and verification of a histone deacetylases-related prognostic signature model for colon cancer
Source: Sci Rep. 2024 Apr 18;14:8983. doi: 10.1038/s41598-024-59724-x (PMC11026370; doi:10.1038/s41598-024-59724-x)
Supplement: Supplementary file 3 — Supplementary Table S1. [file 41598_2024_59724_MOESM3_ESM.docx]

**Table S1.** siRNA sequences for BRD3.

| Gene | sense 5’-3’ | antisense 5’-3’ |
| --- | --- | --- |
| si-NC | CAGAAGAATGGTACAAATCCAAG | CTTCGTTCAGTATGTTAATCGT |
| si-BRD3-1 | CACAGATGACATAGTGCTAAT | TAGCACTATGTCATCTGTGGG |
| si-BRD3-2 | GGGAGATGCTATCCAAGAAGC | TTCTTGGATAGCATCTCCCTG |
| si-BRD3-3 | CCATGTTTACAAATTGTTACA | TAACAATTTGTAAACATGGTG |
