# Supplementary material for: Construction and verification of a histone deacetylases-related prognostic signature model for colon cancer
Source: Sci Rep. 2024 Apr 18;14:8983. doi: 10.1038/s41598-024-59724-x (PMC11026370; doi:10.1038/s41598-024-59724-x)
Supplement: Supplementary file 4 — Supplementary Table S2. [file 41598_2024_59724_MOESM4_ESM.docx]

**Table S2.** Primers for qRT-PCR.

| Gene | Forward | Reverse |
| --- | --- | --- |
| BRD3 | GCATCTGCCTCCTACGACTC | CTGGCGCTTTTCATCGTAGC |
| GAPDH | GGAGCGAGATCCCTCCAAAAT | GGCTGTTGTCATACTTCTCATGG |
